# Single‐cell sequencing reveals alterations in the peripheral blood mononuclear cell landscape and monocyte status during colorectal adenocarcinoma formation

**DOI:** 10.1002/ctm2.1609

**Published:** 2024-03-15

**Authors:** Jiasheng Xu, Yeting Hu, Jie Zhao, Xiangxing Kong, Sijian Xia, Siqi Dai, Lei Ding, Tongtong Bu, Yue Cao, Manjiao Liu, Linlin Yan, Qian Xiao, Hao Guo, Ying Yuan, Dong Xu, Kefeng Ding

**Affiliations:** ^1^ Department of Colorectal Surgery The Second Hospital of Zhejiang University School of Medicine (Key Laboratory of Cancer Prevention and Intervention, China National Ministry of Education, and Key Laboratory of Medical Molecular Biology) Zhejiang University Hangzhou China; ^2^ Zhejiang University Cancer Institute Hangzhou China; ^3^ The State Key Laboratory of Neurology and Oncology Drug Development Jiangsu Simcere Diagnostics Co., Ltd., Nanjing Simcere Medical Laboratory Science Co., Ltd. Nanjing China; ^4^ The Department of Medical Oncology the Second Affiliated Hospital of Zhejiang University Hangzhou China

Dear Editor,

Since colorectal cancer (CRC) is the third most commonly diagnosed cancer and the second leading cause of cancer death globally,^1^, ^2^ we want to explore non‐invasive early diagnosis methods and find some supplements to colonoscopy. In this study, we for the first time performed peripheral blood mononuclear cell (PBMC) transcriptome analysis of healthy individuals, colon adenoma patients and colon adenocarcinoma patients using single‐cell RNA sequencing, and focused on peripheral circulating monocytes changing from healthy people to adenoma patients to carcinoma patients.

Many studies proved both locally infiltrated and circulating immune cells play an important role and change significantly during the development of CRC.^3^, ^4^, ^5^, ^6^ So we focused on immune cells in PBMC. The PBMC samples for single‐cell RNA sequencing were from five healthy people, five adenoma and five adenocarcinoma patients who met the inclusion and exclusion criteria of endoscopic and pathological results (Figure 1A). The detailed methods of this study can be found in the Supporting Information. After quality control, a total of 110 916 cells from 15 samples were filtered, followed by unsupervised clustering and annotation (Figure 1B). Cell types and marker expression are shown by tSNE plots in Figure S1A,B. The genes shown on the dot plot were selected according to high expression in each cluster and canonical markers (Figure 1C).

**FIGURE 1:**
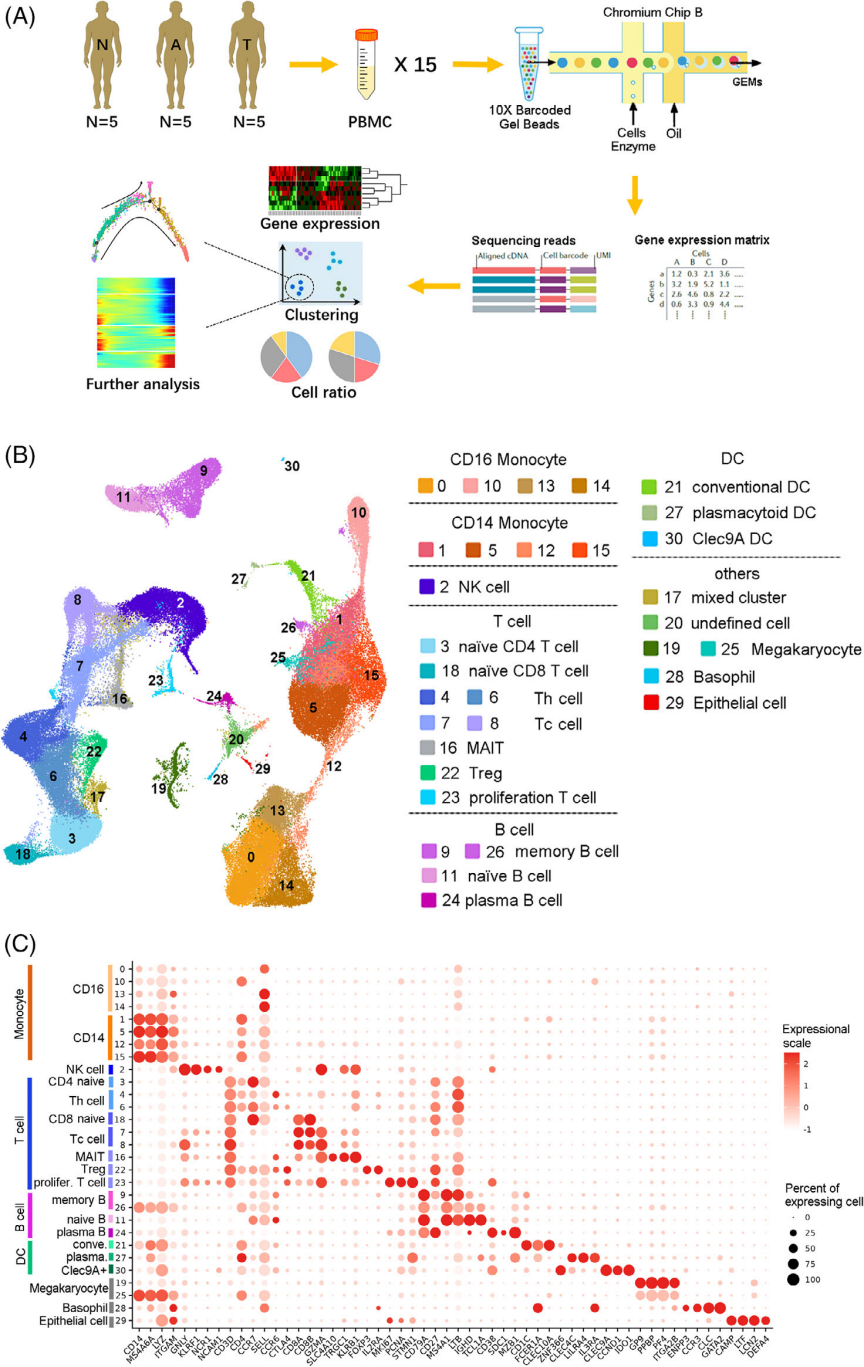
An atlas of peripheral blood mononuclear cells (PBMCs) in healthy people, colon adenoma patients and colon adenocarcinoma patients. (A) Research workflow presenting the overall design of the study. The sample number for each group was 5. N represents normal (healthy) donors, A denotes colon adenoma donors and T denotes colon adenocarcinoma donors. Fifteen PBMC samples from the 15 donors were subjected to 10X Genomics single‐cell transcriptome sequencing. (B) UMAP plot of integrated single‐cell transcriptomes for 110 916 PBMCs. Cells are colour‐coded by clusters generated by graph‐based clustering. Several cell types contain more than one cluster, which are illustrated on the right. Th cells denote T helper cells. Tc cells denote cytotoxic T cells. MAIT denote mucosal‐associated invariant T cells. (C) The dot plot shows selected feature genes for clusters. The left columns display the clusters and their cell type annotations. The expression‐related colour depicts the average expression level, and the dot size depicts the percentage of cells expressing the genes across the cluster population.

We calculated the proportion of the various cell subsets in three groups (Figure S2A). Further comparison with statistical tests showed the proportion of cell types among PBMCs, CD14 monocytes and CD16 monocytes among monocytes and CD8 T cells among T cells in Figure S2B–K. All cell subset ratios across three groups are shown in Figure S2L.

We next performed a cell‐interaction analysis of major immune cell types to explore differences from the perspective of cell‒cell communication. An overall view showed that the number of L‐R pairs displayed a downwards trend in the N/A/T groups, as well as the total interaction strength (Figure 2A). The radar maps indicated CD14 monocyte interaction in tumour patients decreased in all groups except for CD16 monocytes, but CD16 monocyte interactions with various cells increased (Figure 2B). We further counted the number of interactions between the pairwise cell types, and Figure 2C shows the statistical results of the N/A/T three groups separately. The enrichment analysis on L‐R pairs indicated the number of Gene Ontology and Kyoto Encyclopedia of Genes and Genomes (KEGG) terms enriched all decreased sequentially in the N/A/T groups (Figure 2D). Next, the top 20 KEGG‐enriched pathways were examined, and three important immune‐related pathways were marked in red (Figure 2E).

**FIGURE 2:**
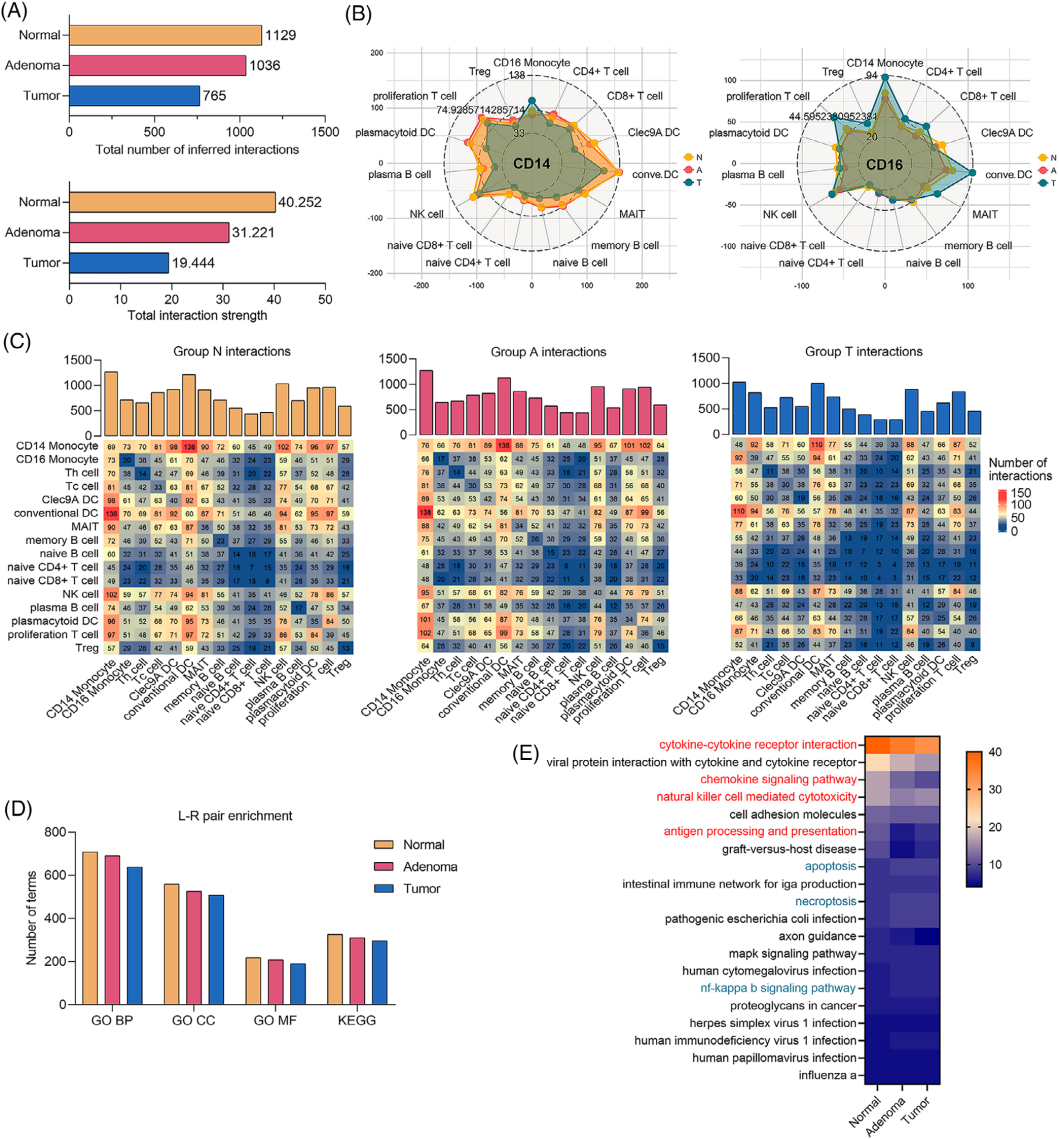
Comprehensive insights into differences in cell interactions among peripheral blood mononuclear cells (PBMCs) from different pathological states. (A) Total cell interaction numbers (upper panel) and total cell interaction strength (lower panel) in PBMCs from the normal group, adenoma group and tumour group. (B) The left radar graph shows the number of interactions between CD14 monocytes (source) and other major cell types (target). The right radar graph shows the number of interactions between CD16 monocytes (source) and other major cell types (target). (C) Heatmaps of the three groups display the interaction numbers between each other of the cell types listed in the left column and the bottom. The bar graphs on the top show the overall interaction numbers for each cell type. (D) The total number of enriched Gene Ontology (GO) terms and Kyoto Encyclopedia of Genes and Genomes (KEGG) terms for each group. (E) Top 20 terms selected from enriched KEGG terms present in all three groups. The terms that are related to immune cell interactions and varied across the three groups are marked in red. Cell interactions were analysed using CellPhoneDB.

We next focused on the L‐R pairs in three immune‐related pathways from or to CD14 and CD16 monocytes and drew a bubble chart to show their expression levels (Figure S3A,B). Sankey plots revealed selected interaction pairs that showed significant changes between the two compared groups (Figure S4A), and these pairs were selected from the bubble chart. Then, we summarized the characteristics of the above cell communications (Figure S4B). First, the interaction of tumour necrosis factor family molecules in adenoma patients is mainly produced by CD14 and acts on CD16 and classical DCs. However, in tumour patients, CD16 produces ligands that act on CD14 and classical DCs. Second, chemokine interactions mainly exist among peripheral immune cells of adenoma patients. Third, some leukocyte antigen‐mediated antigen presentation is enhanced in tumour patients, and multiple kinds of cells present antigens to natural killer and cytotoxic T cells.

Then, monocytes were performed unsupervised clustering and artificial annotation based on high‐expressed genes (Figure 3A). The classical monocytes are mostly derived from tumour patients and granulocyte‐like monocytes related to innate immune mostly derived from healthy donors (Figure 3B). The trajectories of these monocytes were inferred and marked as group sources (Figure 3C). The cells on the trajectory were divided into three states (Figure 3D). Along the sequence of normal‐adenoma‐tumour, the proportion of state 1 monocytes gradually decreased, the proportion of state 2 monocytes gradually increased, and state 3 had the highest proportion of monocytes in tumour patients (Figure 3E). To further resolve the subpopulations of monocytes in States 1–3, we annotated the trajectory of cells with cell subpopulations (Figure 3F) and then counted the proportion of each subpopulation in States 1, 2 and 3 (Figure 3G). In addition, the tracks labelled ‘Pseudotime’ showed that the paths of State 1–State 2 or State 3 are defined as two cell fates (Figure 3H). Heatmaps show the dynamic changes in gene expression along the two cell fates and these genes were divided into four groups. The top‐panel group genes were mainly involved in the innate immune response. In contrast, MHCII molecules in group 3 and group 4 genes increased expression along pseudotime paths, such as HLA‐DRB1 and HLA‐DPA1, which are mainly related to specific immune responses (Figure 3I).

**FIGURE 3:**
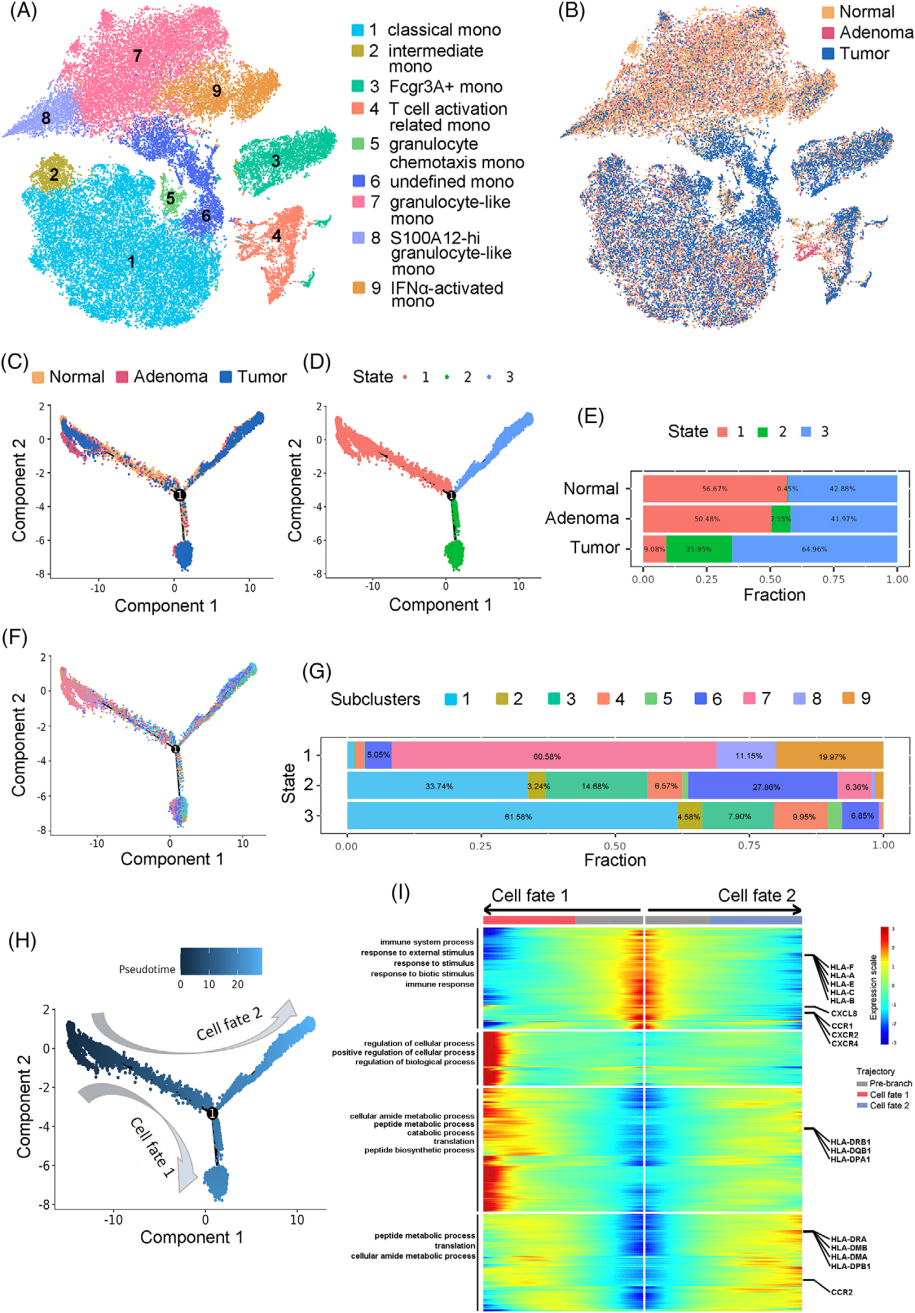
Trajectory analysis of monocyte transition states across the three groups. (A) The tSNE (t‐Distributed Stochastic Neighbor Embedding) plot displays the result of monocyte subclustering, and the subtypes are labelled in different colours. Cell‐type annotations are provided on the right. (B) The tSNE projection showing monocytes from three groups; colour coding is according to disease state. (C) Trajectory plot of monocytes. Cells from three groups of different disease states are labelled by colour. (D) Cells on the trajectory are divided into three transition states. (E) The proportion of three cell states on the trajectory in each disease state group. (F) Cells on the trajectory belonging to different monocyte subclusters are labelled by colours consistent with (A). (G) The proportion of nine monocyte subclusters in each cell state on the trajectory. (H) Pseudotime order of cells on the trajectory. The results indicate two paths of cell transition fate. (I) Heatmap showing dynamic changes in gene expression along the two cell fates according to (H). Cell fate 1 (left panel heatmap) and cell fate 2 (right panel heatmap). Significantly changed genes along the trajectory are clustered into four groups and labelled by colour (leftmost column).

The expressional levels in classical monocytes of indicated genes on the heatmap (Figure 3I) were presented by dot plot (Figure 4A). The two highlighted genes were concurrently included in differentially expressing genes between groups in classical monocytes. Another four genes were found both significantly decreased in adenoma versus normal individuals and in tumour versus adenoma patients (Figure 4B). The expression of the aforementioned genes in CD14‐positive cells is displayed in Figure 4C. NAMPT, BCL2A1 and TREM1 expressed in more than 25% of CD14‐positive cells with decreasing expression along normal‐adenoma‐tumor groups. The tSNE plot showed CD14 expression and the cells expressing both CD14 and BCL2A1 (Figure 4D,E). At the end, we calculated the ratio of double‐positive (NAMPT+ /BCL2A1+ /TREM1+, CD14+) PBMCs to CD14‐positive PBMCs (Figure 4F) and the ratio of double‐positive PBMCs to total PBMCs (Figure 4G) in three groups. The statistical results indicated the ratio of double‐positive PBMCs to CD14‐positive PBMCs had a significant difference between groups (Figure 4H). Finally, Figure 4I presents a graphical abstract of these findings.

**FIGURE 4:**
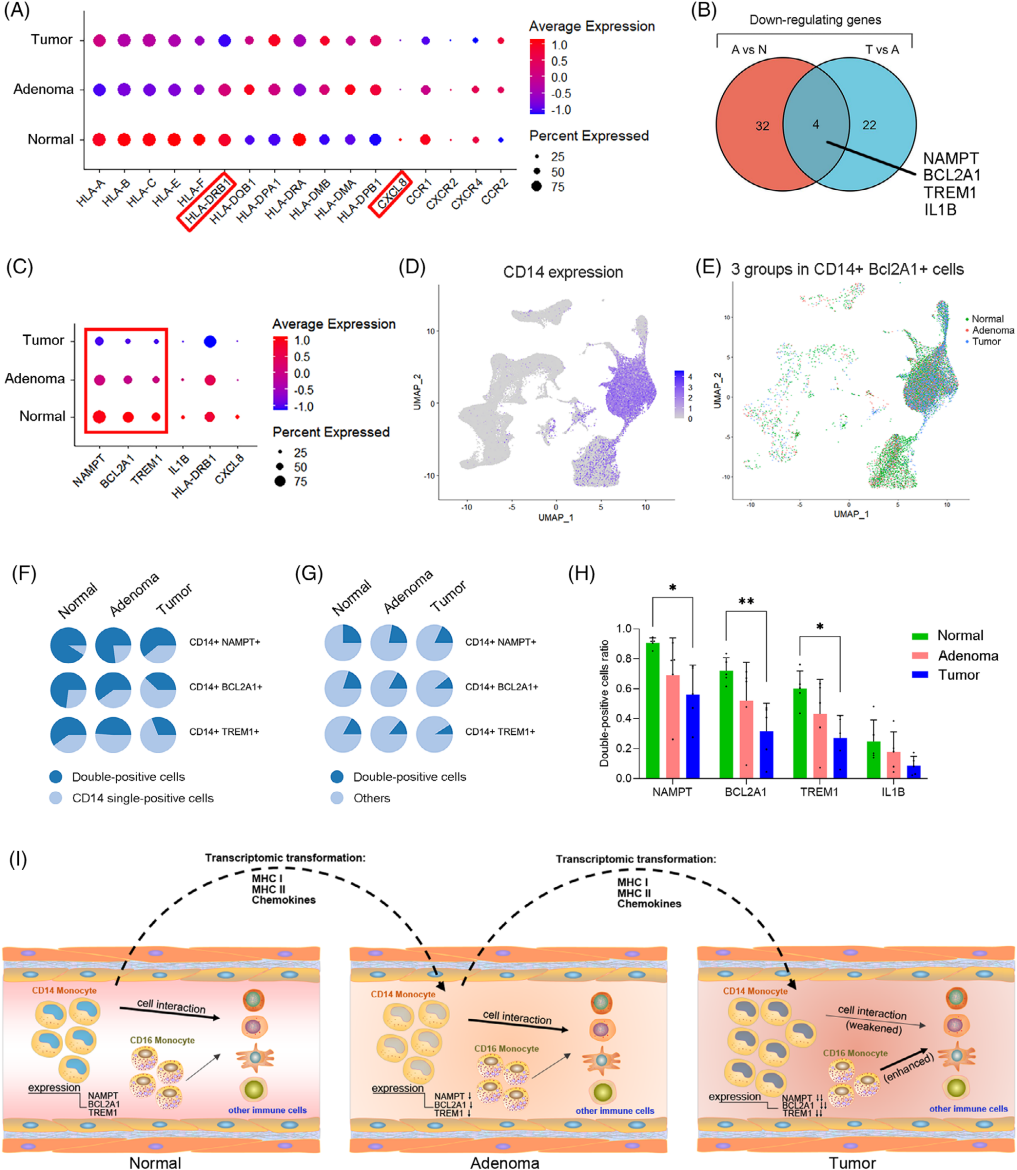
Significantly different genes and cell types across three groups. (A) The dot plot shows the expression of HLA and chemokine genes in classical mono. These genes are labelled in the dynamic‐changing genes of trajectory analysis in Figure 3. The expression‐related colour depicts the average expression level, and the dot size depicts the percentage of cells expressing the genes across each group. Two genes indicated by red boxes are expressed significantly differently across classical mono in three groups. (B) The overlap between genes significantly down‐regulated in adenoma classical mono compared to normal and genes significantly down‐regulated in tumour classical mono compared to adenoma. The significant genes are selected based on the criteria of *p*‐value < .05 and log2FC > 0.5. (C) The dot plot shows the expression of four overlapping genes and the two genes highlighted in (A) across three groups of CD14‐positive cells (cells with CD14 expression > 0 in all peripheral blood mononuclear cell [PBMC]). (D) Feature plot shows CD14 expression in all PBMCs. (E) Cells with CD14 expression > 0 and Bcl2A1 expression > 0 labelled by three groups. (F) The proportion of double‐positive cells to CD14‐positive cells across the three groups. (G) The proportion of double‐positive cells to all PBMCs across the three groups. (H) The statistical results of the proportion of double‐positive cells to CD14‐positive cells among 15 samples in the three groups. *, *p*‐value < .05. **, *p*‐value < .01. (I) The graphical abstract of the study. The graph presented immune cells in blood vessels of normal people, colon adenoma patients and colon tumour patients. The transcriptomic transformations from normal to adenoma to tumour were presented in the expression levels of MHC I, MHC II and chemokines. The cell interactions and the gene expressions in CD14 monocytes also presented changes in different disease statuses.

In conclusion, our study first presents a landscape of peripheral immune cells in different colon neoplastic disease states at single‐cell transcriptomic levels. Monocyte analysis revealed cell‐status transformation trends along the non‐neoplasm to adenoma to cancer transition. The expression of NAMPT, BCL2A1 and TREM1 in peripheral classical monocytes decreased during colorectal adenocarcinoma formation. Although further validation is required, these results provide evidence for novel CRC diagnostic biomarker findings.

## AUTHOR CONTRIBUTIONS

Jiasheng Xu, Yeting Hu and Jie Zhao performed the experiment, analyzed data and wrote the manuscript. Siqi Dai, Xiangxing Kong and Lei Ding helped to write the manuscript, search the sample and literature and help with data checking and language polishing. Kefeng Ding designed the study and reviewed the manuscript.

## CONFLICT OF INTEREST STATEMENT

There authors declare no conflict of interest.

## FUNDING INFORMATION

The Key R&D Program of Zhejiang (2023C03049), The National Natural Science Foundation of China (82103684, 11932017 and 82072624), the National Key R&D Program of China (2017YFC0908200), a project supported by the Scientific Research Fund of Zhejiang University (XY2021025), a project of the regional diagnosis and treatment centre of the Health Planning Committee (No. JBZX‐201903 to Kefeng Ding) and the Fundamental Research Funds for the Central Universities (No. 226‐2022‐00009) to Kefeng Ding.

## ETHICS STATEMENT

This study was conducted in accordance with the recommendations of the Ethics Committee of the Second Affiliated Hospital of Zhejiang University(2021‐LYS‐0473). The protocol was adopted by the Ethics Committee of the Second Affiliated Hospital of Zhejiang University. All subjects gave written informed consent in accordance with the Declaration of Helsinki.

## Supporting information


**FIGURE S1 Single‐cell RNA sequencing (scRNA‐seq) profiling by tSNE** **plot. (A)** The tSNE plot of integrated single‐cell transcriptomes and results of cell annotation of 110 916 PBMCs revealed by scRNA‐seq. The cluster colours and cell type annotations are consistent with Figure 1B. Th cells denote T helper cells. Tc cells denote cytotoxic T cells. MAIT denote mucosal‐associated invariant T cells. **(B)** tSNE feature plots showing expression of selected canonical marker genes for defined cell types. CD3D, a marker of T cells. CD79A, a marker of B cells. CD14, a marker of CD14 monocytes. CSF3R, a marker of monocytes or myeloid cells. CCR7, marker of naïve T cells. GZMA, marker of NK cells and cytotoxic T cells.

**FIGURE S2 Comparison of major cell type ratios across the three groups revealed by single‐cell RNA sequencing (scRNA‐seq). (A)** Relative abundance of major cell types in the three groups. The relative percentage of each cell type is the mean value of five samples in each group. **(B, C, F, G, H, I and J)** Percentage of CD14 monocytes **(B)**, CD16 monocytes **(C)**, NK cells **(F)**, CD4+ T cells **(G)**, CD8+ T cells **(H)**, DCs **(I)** and B cells **(J)** among PBMCs in the three groups. **(D, E)** Percentage of CD14 monocytes and CD16 monocytes among monocytes in the three groups. **(K)** Percentage of CD8+ T cells among T cells in the three groups. The spot represents each sample result. The Wilcoxon test was used to assess statistical significance. *, *p*‐value < .05. **, *p*‐value < .01. CD4+ T cells, including naïve CD4 T cells and Th cells. CD8+ T cells, including naïve CD8 T cells and Tc cells. B cells, including memory B cells, naïve B cells and plasma B cells. DC, including conventional DC, plasmacytoid DC and Clec9A DC. Monocytes, including CD14 monocytes and CD16 monocytes. T cells, including all kinds of T cells present in the previous UMAP (clusters 3, 4, 6, 7, 8, 16, 18, 22 and 23). All of these cell type annotations are consistent with the UMAP and dot plot in Figure 1. **(L)** Statistical histogram for the percentage of cell types among PBMCs. There were five samples in each group. Pairwise comparisons among the three groups were performed, as was a two‐tailed t‐test. *, *p*‐value < .05.

**FIGURE S3 Bubble plot of CD14 and CD16 monocyte‐related interactions in the three groups. (A)** Bubble charts showing the interaction between CD14 monocytes and other cell types based on L‐R pairs in selected Kyoto Encyclopedia of Genes and Genomes (KEGG) pathways, including cytokine‐cytokine receptor interaction, chemokine signalling pathway and antigen processing and presentation. **(B)** Bubble charts showing the interaction between CD16 monocytes and other cell types based on L‐R pairs in selected KEGG pathways, including cytokine‐cytokine receptor interaction, chemokine signalling pathway, antigen processing and presentation. The size of the bubbles indicates the significance of the interaction, and the colour of the bubbles indicates normalized mean expression levels. The values were calculated by CellPhoneDB.

**Figure S4 Differences in cell interactions across different pathological states viewed by L‐R pairs. (A)** Sankey diagram showing significantly changed L‐R pairs with corresponding source‐target cell types in adenoma versus normal (upper panel), tumour versus adenoma (middle panel) and tumour versus normal (lower panel). L‐R pairs were selected according to the bubble plot in the supplementary figure and prepared for statistical analysis. Red lines indicate that the interaction strength increased, and blue lines indicate that the interaction strength decreased. **(B)** Summary illustration depicting potential L‐R pairs between monocytes and other cell types specifically enhanced in the adenoma PBMCs and in the tumour PBMCs. The orientations of arrows represent the target cells that express receptors.

Supporting Information

## Data Availability

All data is available. Please contact us to access if it is needed.

## References

[1] Xi Y , Xu P . Global colorectal cancer burden in 2020 and projections to 2040. Transl Oncol. 2021;14(10):101174. doi:10.1016/j.tranon.2021.101174. Epub 2021 Jul 6. 34243011 PMC8273208

[2] Campos FG . Colorectal cancer in young adults: a difficult challenge. World J Gastroenterol. 2017;23:5041‐5044. 28811701 10.3748/wjg.v23.i28.5041 PMC5537173

[3] Grizzi F , Basso G , Borroni EM , et al. Evolving notions on immune response in colorectal cancer and their implications for biomarker development. Inflamm Res. 2018;67(5):375‐389. doi:10.1007/s00011-017-1128-1 29322204

[4] Fan A , Wang B , Wang X , et al. Immunotherapy in colorectal cancer: current achievements and future perspective. Int J Biol Sci. 2021;17(14):3837‐3849. doi:10.7150/ijbs.64077. Published 2021 Sep 3. 34671202 PMC8495390

[5] Hagland HR , Lea D , Watson MM , Søreide K . Correlation of blood T‐cells to intratumoural density and location of CD3^+^ and CD8^+^ T‐cells in colorectal cancer. Anticancer Res. 2017;37(2):675‐683. doi:10.21873/anticanres.11363 28179316

[6] Waidhauser J , Nerlinger P , Arndt TT , et al. Alterations of circulating lymphocyte subsets in patients with colorectal carcinoma. Cancer Immunol Immunother. 2022;71(8):1937‐1947. doi:10.1007/s00262-021-03127-8. Epub 2021 Dec 20. 34928423 PMC9293872

[7] Zhu L , Yang P , Zhao Y , et al. Single‐cell sequencing of peripheral mononuclear cells reveals distinct immune response landscapes of COVID‐19 and influenza patients. Immunity. 2020;53(3):685‐696. e3. 32783921 10.1016/j.immuni.2020.07.009 PMC7368915

[8] Efremova M , Vento‐Tormo M , Teichmann SA , Vento‐Tormo R . CellPhoneDB: inferring cell‐cell communication from combined expression of multi‐subunit ligand‐receptor complexes. Nat Protoc. 2020;15(4):1484‐1506. 32103204 10.1038/s41596-020-0292-x

[9] Interlandi M , Kerl K , Dugas M . InterCellar enables interactive analysis and exploration of cell‐cell communication in single‐cell transcriptomic data. Commun Biol. 2022;5(1):21. 35017628 10.1038/s42003-021-02986-2 PMC8752611

[10] Qiu X , Mao Q , Tang Y , et al. Reversed graph embedding resolves complex single‐cell trajectories. Nat Methods. 2017;14(10):979‐982. 28825705 10.1038/nmeth.4402 PMC5764547

