# Supplementary figures and images for: Single‐cell sequencing reveals alterations in the peripheral blood mononuclear cell landscape and monocyte status during colorectal adenocarcinoma formation
Source: Clin Transl Med. 2024 Mar 15;14(3):e1609. doi: 10.1002/ctm2.1609 (PMC10941536; doi:10.1002/ctm2.1609)

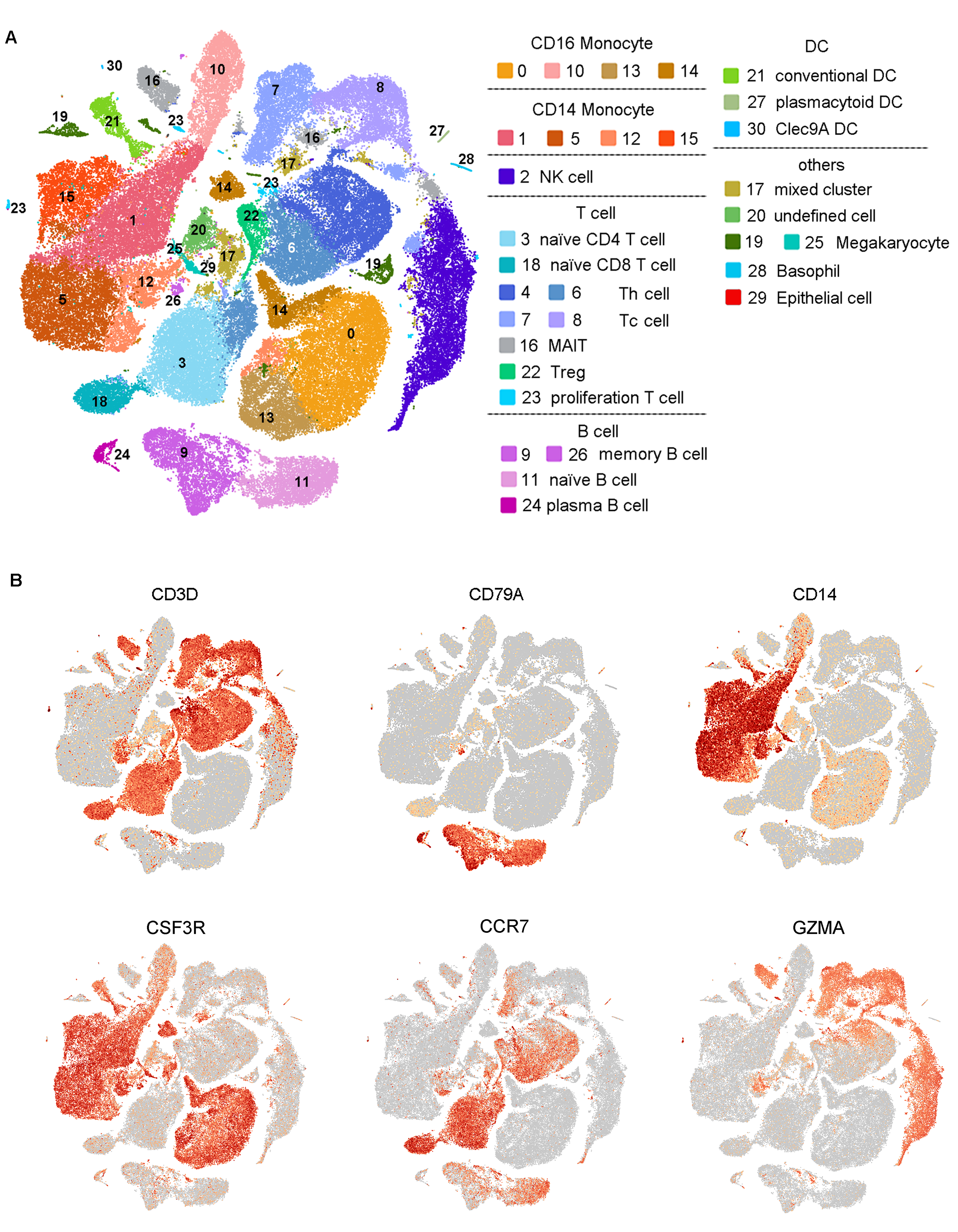

Supplement: Supplementary file 1 — FIGURE S1 Single‐cell RNA sequencing (scRNA‐seq) profiling by tSNE plot. (A) The tSNE plot of integrated single‐cell transcriptomes and results of cell annotation of 110 916 PBMCs revealed by scRNA‐seq. The cluster colours and cell type annotations are consistent with Figure 1B. Th cells denote T helper cells. Tc cells denote cytotoxic T cells. MAIT denote mucosal‐associated invariant T cells. (B) tSNE feature plots showing expression of selected canonical marker genes for defined cell types. CD3D, a marker of T cells. CD79A, a marker of B cells. CD14, a marker of CD14 monocytes. CSF3R, a marker of monocytes or myeloid cells. CCR7, marker of naïve T cells. GZMA, marker of NK cells and cytotoxic T cells. [file CTM2-14-e1609-s003.tif]

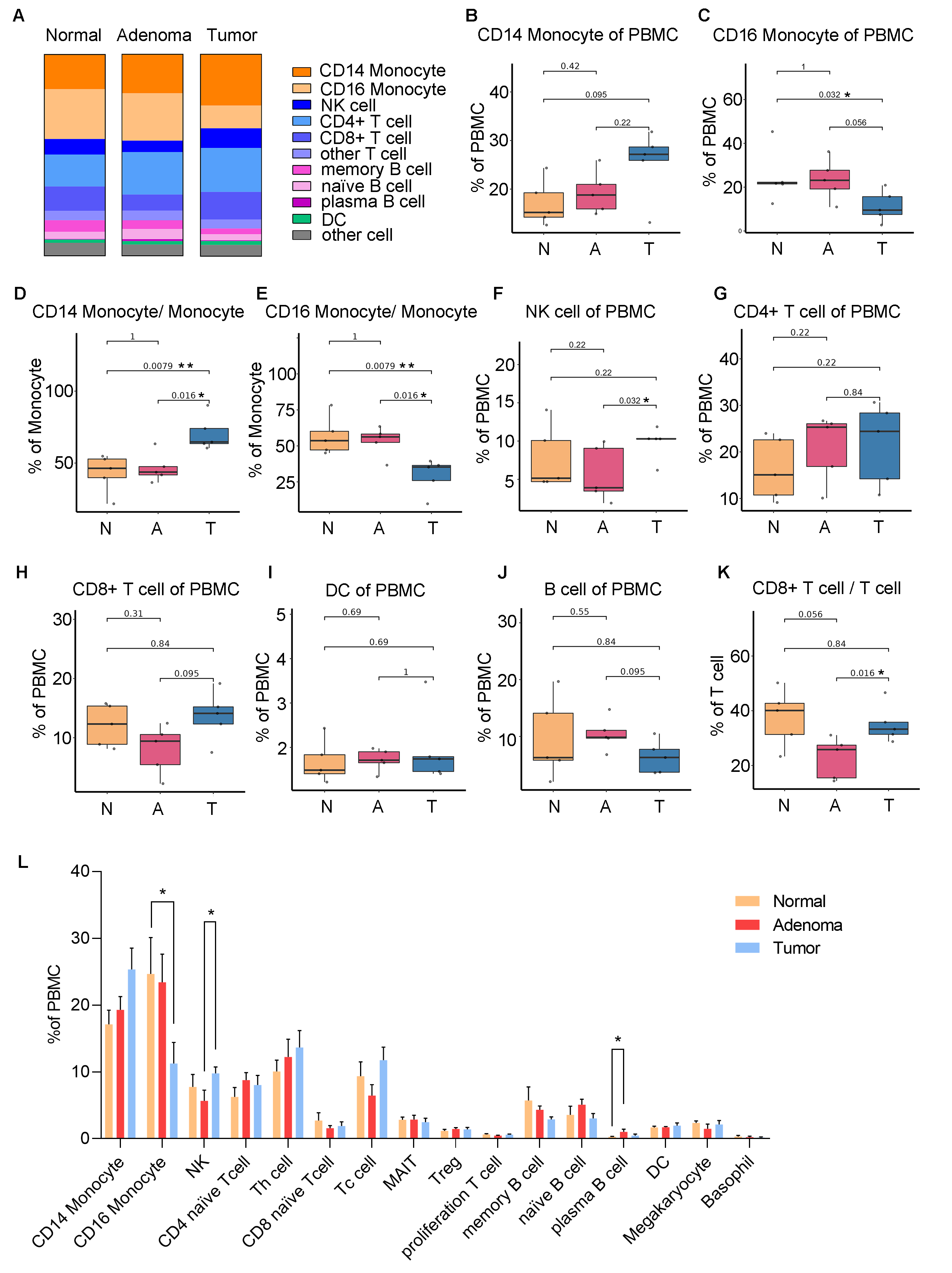

Supplement: Supplementary file 2 — FIGURE S2 Comparison of major cell type ratios across the three groups revealed by single‐cell RNA sequencing (scRNA‐seq). (A) Relative abundance of major cell types in the three groups. The relative percentage of each cell type is the mean value of five samples in each group. (B, C, F, G, H, I and J) Percentage of CD14 monocytes (B), CD16 monocytes (C), NK cells (F), CD4+ T cells (G), CD8+ T cells (H), DCs (I) and B cells (J) among PBMCs in the three groups. (D, E) Percentage of CD14 monocytes and CD16 monocytes among monocytes in the three groups. (K) Percentage of CD8+ T cells among T cells in the three groups. The spot represents each sample result. The Wilcoxon test was used to assess statistical significance. *, p‐value < .05. **, p‐value < .01. CD4+ T cells, including naïve CD4 T cells and Th cells. CD8+ T cells, including naïve CD8 T cells and Tc cells. B cells, including memory B cells, naïve B cells and plasma B cells. DC, including conventional DC, plasmacytoid DC and Clec9A DC. Monocytes, including CD14 monocytes and CD16 monocytes. T cells, including all kinds of T cells present in the previous UMAP (clusters 3, 4, 6, 7, 8, 16, 18, 22 and 23). All of these cell type annotations are consistent with the UMAP and dot plot in Figure 1. (L) Statistical histogram for the percentage of cell types among PBMCs. There were five samples in each group. Pairwise comparisons among the three groups were performed, as was a two‐tailed t‐test. *, p‐value < .05. [file CTM2-14-e1609-s002.tif]

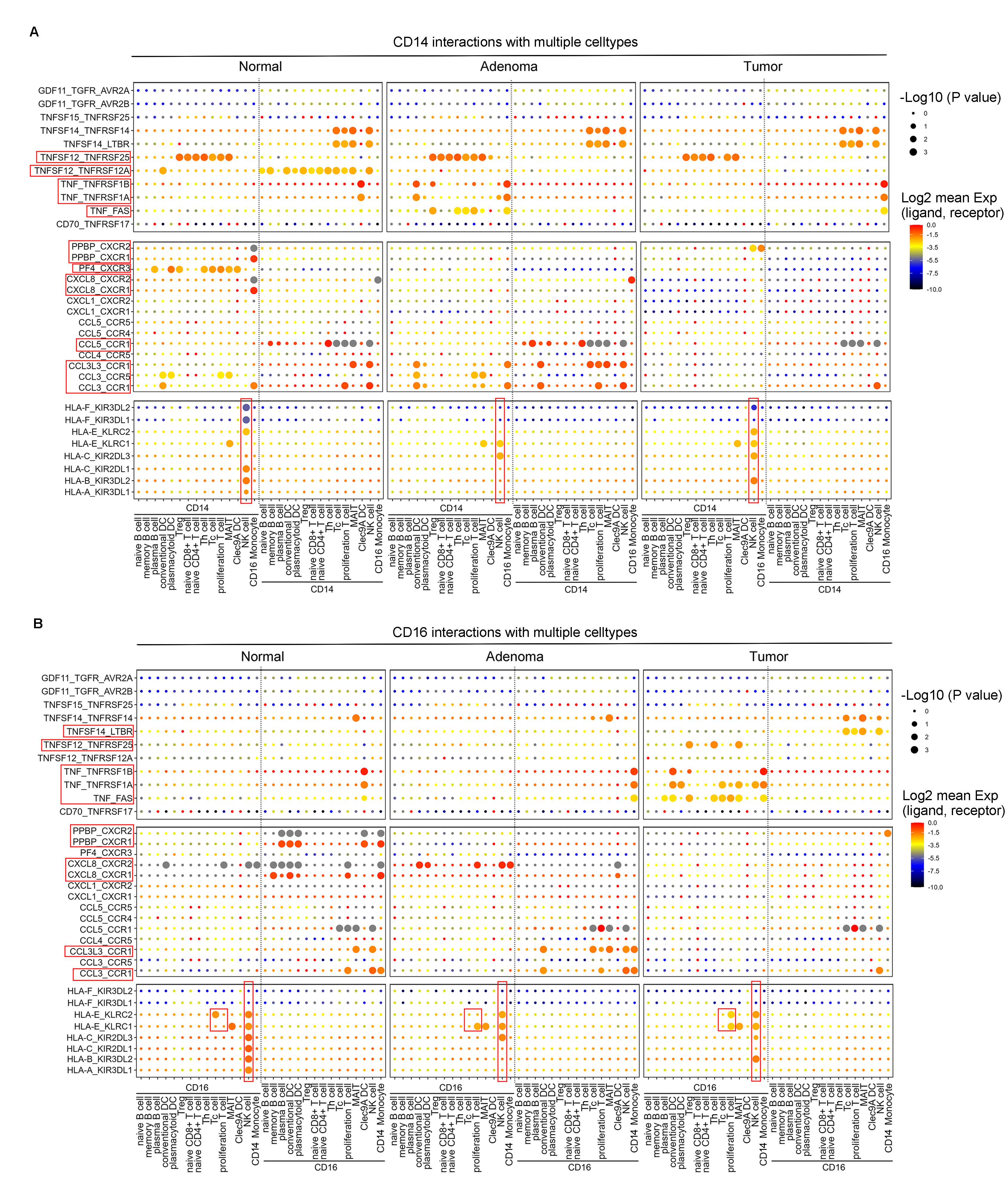

Supplement: Supplementary file 3 — FIGURE S3 Bubble plot of CD14 and CD16 monocyte‐related interactions in the three groups. (A) Bubble charts showing the interaction between CD14 monocytes and other cell types based on L‐R pairs in selected Kyoto Encyclopedia of Genes and Genomes (KEGG) pathways, including cytokine‐cytokine receptor interaction, chemokine signalling pathway and antigen processing and presentation. (B) Bubble charts showing the interaction between CD16 monocytes and other cell types based on L‐R pairs in selected KEGG pathways, including cytokine‐cytokine receptor interaction, chemokine signalling pathway, antigen processing and presentation. The size of the bubbles indicates the significance of the interaction, and the colour of the bubbles indicates normalized mean expression levels. The values were calculated by CellPhoneDB. [file CTM2-14-e1609-s004.tif]

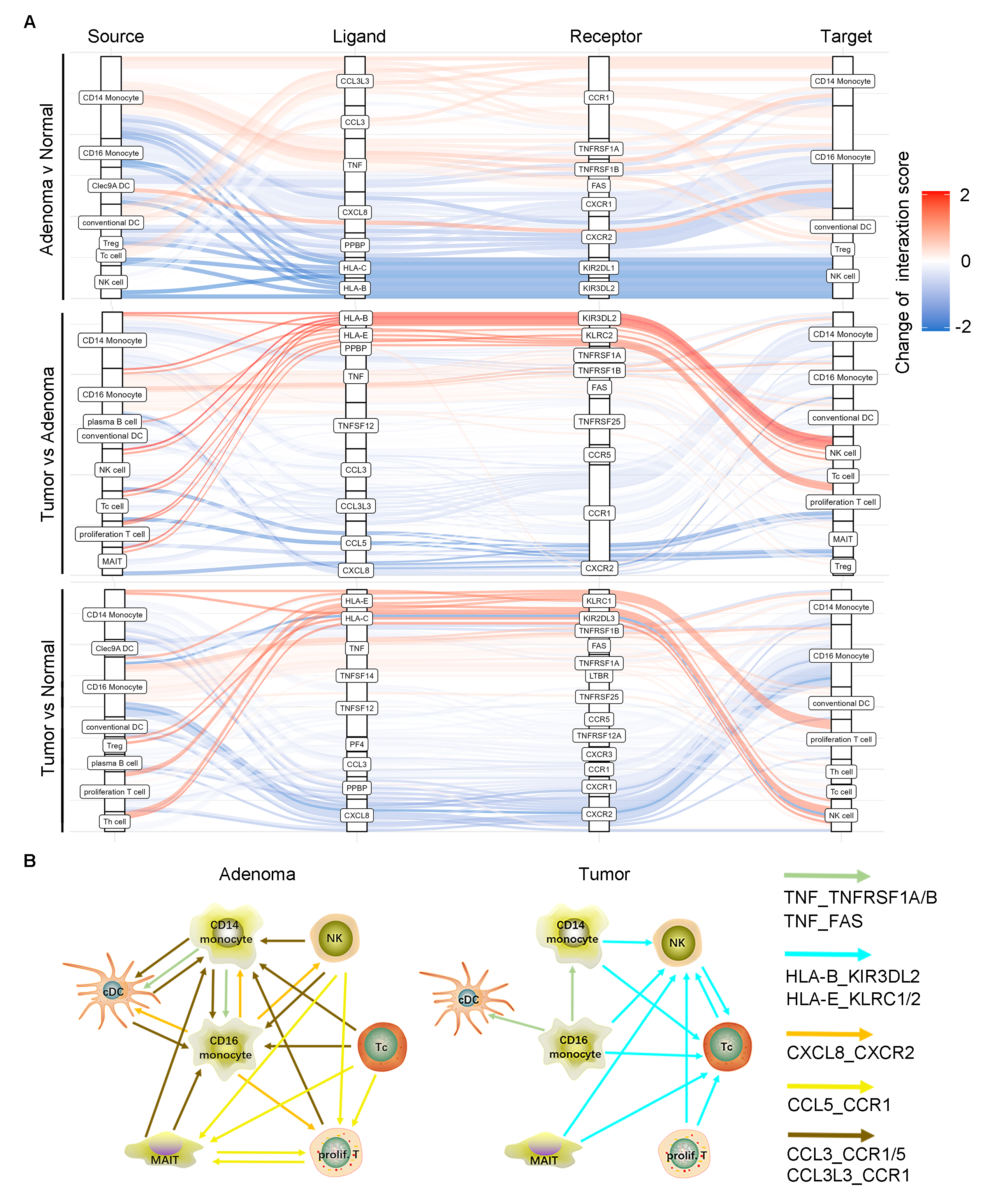

Supplement: Supplementary file 4 — Figure S4 Differences in cell interactions across different pathological states viewed by L‐R pairs. (A) Sankey diagram showing significantly changed L‐R pairs with corresponding source‐target cell types in adenoma versus normal (upper panel), tumour versus adenoma (middle panel) and tumour versus normal (lower panel). L‐R pairs were selected according to the bubble plot in the supplementary figure and prepared for statistical analysis. Red lines indicate that the interaction strength increased, and blue lines indicate that the interaction strength decreased. (B) Summary illustration depicting potential L‐R pairs between monocytes and other cell types specifically enhanced in the adenoma PBMCs and in the tumour PBMCs. The orientations of arrows represent the target cells that express receptors. [file CTM2-14-e1609-s001.tif]
