# Supplementary material for: Single‐cell sequencing reveals alterations in the peripheral blood mononuclear cell landscape and monocyte status during colorectal adenocarcinoma formation
Source: Clin Transl Med. 2024 Mar 15;14(3):e1609. doi: 10.1002/ctm2.1609 (PMC10941536; doi:10.1002/ctm2.1609)
Supplement: Supplementary file 5 — Supporting Information [file CTM2-14-e1609-s005.docx]

**Supplemental Methods**

**1 Human subjects**

All donors were recruited from the Second Affiliated Hospital Zhejiang University School of Medicine between October 2021 and February 2022. The study was designed to detect the characteristics of colonic adenoma and colon adenocarcinoma at the PBMC transcriptome level. Blood samples from three groups, 5 healthy people without adenoma or carcinoma, 5 patients with only colonic adenoma, and 5 patients with only colonic carcinoma, were collected for further single-cell RNA-seq in this study. All of the donors included in the study were verified by colonoscopy as were the healthy individuals. Among the adenoma patients, the possibility of FAP was excluded. In total, 3 ml blood was collected from each donor, and all of the blood samples were sent to the laboratory of Jiangsu Simcere Diagnostics Co., Ltd. for single-cell preparation within 4 hours of collection.

**2 Single-cell preparation for scRNA-seq**

Fresh whole blood samples (2 ml) were added to a centrifuge tube, and the same volume of 1X DPBS was added. After gentle mixing, the mixtures were slowly added to 4 ml Ficoll in another centrifuge tube and centrifuged at 300 × g for 20 min at 20°C. PBMCs were collected from the middle layer of the density gradient. Then, we transferred the PBMCs to 10 ml PBS and mixed the cell suspension gently. If the PBMC layer contained some erythrocytes, a lysis step using RBC lysis buffer (Roche) was included, and the cells were washed again. Finally, we added PBS of the appropriate volume to resuspend the PBMCs and calculated cell numbers.

The isolated PBMC samples met the quality standard of total cell number>10000 and cell viability exceeding 85%. The cell suspension was maintained at a concentration of 500-1000 cells/μl, and all cells were independent without adhesion. Cell fragments >40 μM and cell-free nucleic acid were removed from the suspension [7].

**3 Single-cell capture, transcriptome library construction and sequencing**

A 10X Genomics microfluidic system was utilized for single-cell capture. Chromium Single Cell reagent kit was used according to the instructions (10X Genomics) for single-cell capture and transcriptome library construction. The constructed libraries were sequenced in PE150 mode (Pair-End for 150 bp reads) using the NovaSeq platform (Illumina).

**4 Single-cell RNA-seq data processing and analysis**

Raw single-cell sequencing data were generated into feature-barcode matrices through the Cell Ranger pipeline with default parameters. Then, the R package Seurat was utilized for quality control and subsequent analysis, including normalization, batch effect removal, clustering, dimensional reduction and visualization. Raw gene expression matrices were input into R and converted to Seurat objects. We retained high-quality single cells with < 15% mitochondrial reads and < 5% HBA/HBB gene reads. Ultimately, 110916 cells remained for further analysis, and their gene expression matrix was normalized to the total cellular UMI count. The top 2000 most variable genes were selected as features for further dimensional reduction and cluster analysis. Canonical correlation analysis (CCA) was applied to remove batch effects. To reduce dimensionality, PCA was performed based on highly variable genes after scaling the data with respect to UMI counts. Then, the tSNE and UMAP algorithms were utilized to further reduce dimensionality. Shared nearest neighbour graph-based clustering was performed to identify cell clusters.

Differential expression (DE) was assessed using the FindAllMarkers function in Seurat with the Wilcoxon test following the criteria log2-fold change > 0.25, min. pct > 0.25, Bonferroni-corrected P values < 0.05. Cluster marker genes were identified by applying DE analysis for upregulated genes between cells in one cluster and all other clusters in the dataset. Feature plots, violin plots and heatmaps were utilized to illustrate the expression results. The top ranked genes (by log2FC) from each cluster and some classical cell-type markers were comprehensively considered for cluster annotation and cell-type feature gene determination, as displayed in a heatmap. The different cell type ratios were then calculated for each donor, and significant differences between groups were assessed by the Wilcoxon test, in which a P value < 0.05 indicated significance.

**5 Cell interaction analysis**

We applied the CellPhoneDB algorithm to identify cell communication between multiple kinds of cell types using ligand/receptor as defined in the database [8]. Ligand expression in one cell type (source) and the corresponding receptor expression in another cell type (target) were evaluated to determine interaction between cells and to assess the interaction strength. The CellPhoneDB results were input into the visualization software InterCellar [9]. We focused on interactions between CD14/16 monocytes and other cell types through this interactive analysis software. Then, we concentrated on the ligand‒receptor pairs enriched in CD14/16 monocyte-other cell type interactions with high significance (P value < 0.05) compared pairwise in the healthy, adenoma and tumour groups.

**6 Trajectory analysis**

We applied Monocle2 [10] to infer the cell lineage trajectory of monocytes. All of the monocytes were included in the trajectory and ordered according to the progression of tumorigenesis based on the top genes expressed in normal (healthy), adenoma, and tumour-derived monocytes. We performed subpopulation segmentation using unsupervised clustering based on a graph-based algorithm for all monocytes (including clusters 1, 5, 12, 15, 0, 10, 13, and 14 in Figure 1B). Subsequent annotation was performed artificially according to differentially expressed genes (DEGs) in subclusters and DEG enrichment analysis. Then, the cells on the trajectory were labelled by the subcluster cell type. Finally, we applied the ‘plot_genes_branched_pseudotime’ function in the software package to plot gene expression variation along the trajectory.
